# How to Get There When You Are There Already? Defining Presence in Virtual Reality and the Importance of Perceived Realism

**DOI:** 10.3389/fpsyg.2021.628298

**Published:** 2021-05-06

**Authors:** Stefan Weber, David Weibel, Fred W. Mast

**Affiliations:** ^1^Department of Psychology, University of Bern, Bern, Switzerland; ^2^Faculty of Psychology, Swiss Distance University Institute, Brig, Switzerland

**Keywords:** presence, perceived realism, virtual reality, immersion, absorption

## Introduction

The aim of the current opinion paper is to challenge the current definition of presence in the context of virtual reality (VR). Opticians do not only measure visual acuity but also the visual field, stereoscopic vision, and color vision. In the same vein, presence researchers are encouraged to not only measure the experience of “being there” in the sense of attentional allocation to the virtual environment (VE), but also the *perceived realism* of the VE. Perceived realism is the result of an evaluation of the virtual world regarding (1) the subjective degree of reality of the depicted environment and (2) its overall plausibility and credibility. Thus, the sense of presence in a VE is conceived as a composite of being there *and* perceived realism. When in VR, a user will inevitably compare the look of virtual objects to real-world objects and judge the level of congruence (Sutcliffe and Gault, 2004). The user evaluates the plausibility and naturalness of the depicted world as well as the ease of interaction within the VE by answering questions such as: is there a shadow cast? Are the proportions of objects correct? Does the environment correspond to my own movements? Does my virtual body match the proportions of my real body? Just like the visual features, a story and its characters are also evaluated in terms of consistency and plausibility (Park et al., 2010; Gorini et al., 2011): are the consequences of actions plausible? Is the story coherent in itself? Does the causal sequence of events make sense? The answers to these questions define the degree of perceived realism. Perceived realism leads to the experience that a user not only feels surrounded by the VE, but rather has a compelling sense of reality and in extreme cases even forgets that he or she is wearing a head-mounted display (HMD).

Previous papers on presence are based on the assumption that realism enhances presence (e.g., Heeter, 1992; Welch et al., 1996; Lombard and Ditton, 1997; Bystrom et al., 1999) suggested a conceptualization of presence as the degree to which a medium seems realistic. Interestingly, perceived realism is nevertheless not part of the most widely used presence definitions. It is either a possible trigger of presence or is blended in with the term being there. The conceptualization of presence as the experience of being there in a mediated environment dominates current presence definitions. Being there is strongly associated with attentional allocation and the sensation of being surrounded and absorbed by a mediated world. However, we claim that presence in VR requires much more than just being there. With the widespread use of immersive VR technology, it has become an easy task to absorb users in a VE. Thus, judgments about the realism of the VE become increasingly important. Being there and perceived realism are both important but yet different aspects of presence. They need to be combined in order to (1) adequately describe and define the experience of presence and (2) to obtain an appropriate and more complete assessment of presence in VR. Thus, theories and measures of presence need to be extended and establish perceived realism as an important domain besides being there.

## Presence in VR as a Two-Dimensional Construct: Being There and Perceived Realism

### Existing Definitions of Presence

Presence is a shortened term for *telepresence* and was first introduced by Minsky (1980). *Presence* is defined as the sense of being in a mediated environment (Steuer, 1992; Draper et al., 1998) or being in a computer-generated world such as in VR (Sheridan, 1992; Slater and Wilbur, 1997). The sensation can thus be described as the sense of being spatially present at remote places displayed by technical interfaces (Weibel and Wissmath, 2011). Since the concept was first mentioned over 40 years ago, the goal of achieving presence has been considered a crucial if not the most crucial aspect of a successful VR experience (Weech et al., 2019). Several review articles exist which all address the history of presence and its various definitions (McMahan, 2003; Lee, 2004; Hartmann et al., 2015b; Nilsson et al., 2016; Skarbez et al., 2017; Curran, 2018). In the following, we restrict ourselves to the most influential definitions of presence and their commonalities.

According to Steuer (1992), presence is the extent to which one's attention is allocated to the mediated environment rather than to the immediate physical environment. Even though, it has been a challenge for researchers to develop a widely accepted description of the phenomenon (cf. Grassini and Laumann, 2020), a consensus has been established regarding the definition, and the term *being there* is widely used to describe presence (Sheridan, 1992; Steuer, 1992; Witmer and Singer, 1998; Skarbez et al., 2017; Weech et al., 2019). Presence is, thus, most commonly described as a subjective experience of being bodily or physically located in a mediated environment (cf. Hartmann et al., 2015b). Beyond these commonalities, the understanding of presence varies mainly in one point: Some researchers point out the importance of agency and suggest that it is not only the feeling of being there that constitutes presence, but also the feeling of being able to interact with the VE. Schubert et al. (1999) propose that presence arises when the VE offers opportunities to actively act within the mediated environment. In line with this assumption, Sanchez-Vives and Slater (2005) propose that “the sense of “being there” in a VE is grounded on the ability to “do there”” (p. 9). Accordingly, Wirth et al. (2007) suggest that the occurrence of presence not only refers to the sensation of being located inside the mediated environment, but also to the sensation of being able to take action in the mediated environment (e.g., moving objects). Wirth et al. assume that this aspect of presence is particularly important in the context of video games or VR environments, but less important for books and films. Potential actions, however, have not been able to establish themselves as a second dimension of presence besides “being there” and are widely neglected in current studies on presence.

In this opinion article, we focus on Presence in VR or in VEs, respectively. Presence refers to mediated content in general and has thus been applied to various types of media including movies and TV shows (e.g., Kim and Biocca, 1997; Lee, 2004). The medium sets the framework in which presence experiences can occur: previous studies revealed that along with technical progress and increased system immersion (graphical resolution, positional tracking, possibilities of interaction up to VR glasses with a complete enclosure of the user), modern VR settings are able to generate higher presence than traditional media (for an overview see Hartmann et al., 2015b). This sets presence experiences in VR apart. As mentioned above, Wirth et al. (2007) assume that compared to traditional media, the perception of possible action is an important factor within VE. Most commonly, however, the concept of presence is still used independently of the medium and the definition of presence in the context of VR is exactly the same as the definition of presence in other media (e.g., Schuemie et al., 2001). For example, presence was described as a form of being there in film (e.g., Bracken and Skalski, 2010) and in VR (e.g., Slater, 2018). Accordingly, in current studies on VR presence is understood in the sense of “being there” as well. For example, presence is defined as “the extent to which one feels present in the mediated environment, rather than in the immediate physical environment” (Wehden et al., 2021, p. 6), “the extent to which one feels present in the mediated environment” (Yang et al., 2021, p. 2), “the subjective perception of being in a particular place, even if situated physically somewhere else” (Jaalama et al., 2021, p. 2), or as “the subjective feeling of the user of physically being in the virtual environment rather than in the place where the body is located” (Rauscher, 2021, p. 128).

**Figure 1:**
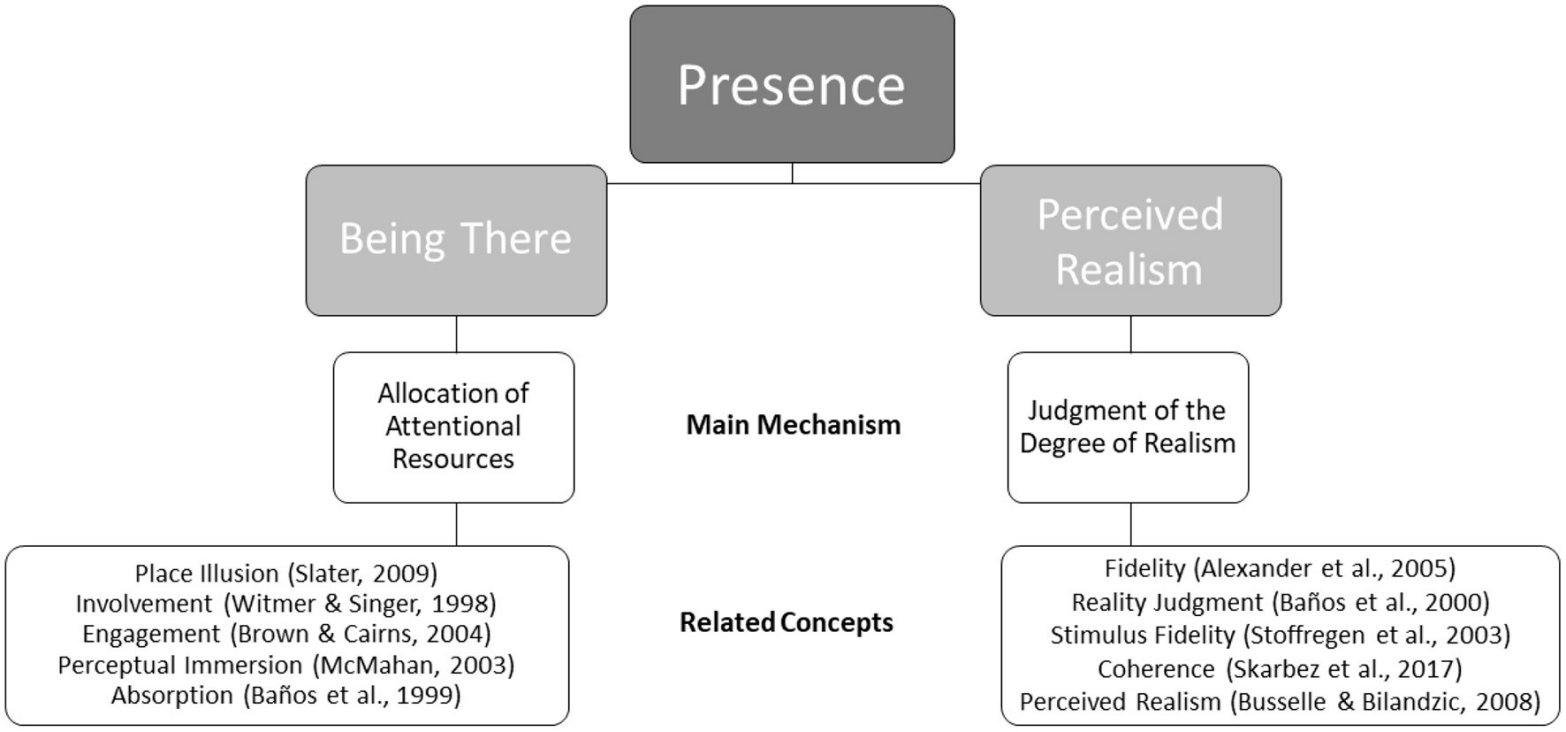
Being there and perceived realism as dimensions of presence.

However, VR is fundamentally different from other media; it is highly immersive and it disconnects the user from the surrounding “real” world. This entails that the conventional definition of presence needs to be refined. In the following, we briefly reflect on existing definitions, explore alternative concepts, and present our own solution for a more complete and contemporary definition of presence. The key elements of our proposed new definition of Presence as a two-dimensional construct are summarized in Figure 1.

### Presence as Being There

Allocating attention is the essential element in many concepts of presence. For example, Lombard and Ditton (1997) use the term *illusion of non-mediation*. This illusion occurs when a person does no longer perceive the mediated environment as being displayed by a media device. According to Slater (2018), it is a *perceptual* illusion, where “the brain-body system automatically and rapidly reacts” (p. 432) to content presented in VR, i.e., it does not require higher-order cognitive processing. A similar concept was proposed by Wirth et al. (2007), according to which presence occurs when attention is allocated to the mediated environment being the user's primary egocentric frame of reference (PEFR). As many authors have pointed out, the sensation of being there depends on the amount of attentional resources allocated to the virtual world rather than to internal thoughts or non-available sensory information (e.g., Baños et al., 1999; Brown and Cairns, 2004; Witmer et al., 2005; for an overview see Skarbez et al., 2017). We argue that this shift of attentional focus is the main concept that underlies the sense of being there.

Accordingly, we define *being there* as the allocation of attentional resources to the mediated world and the sensation of perceptually being surrounded by the VE. Thus, presence is commonly associated with increased attention toward the VE and decreased attention toward external factors and the medium itself. However, presence in this sense could also be achieved by other activities requiring our attention such as focusing on the street while driving a car—clearly not a task where one would use the term presence. We question Slater's binary view (see Slater and Steed, 2000) of a virtual experience as being either real (“I am there”) or not (“I am not there”), based on a probability estimation. Wissmath et al. (2011), who analyzed mental self-localization during a virtual roller-coaster ride, were able to show that self-localization is indeed not tied to one single place: their results revealed that participants could distribute their self-localization in both realities, and the two values added up. This suggests that it is possible to feel located in two places at the same time. A binary view might be true for judgments about reality itself (what we experience can either be considered real or unreal; whereas there are certainly varying degrees of confidence attributed to the latter). However, research participants and players arguably rarely expect a virtual world to reflect the true state of reality. They are always aware of the artificial nature of the virtual scene unless technology improves significantly in the near future (this is in line with Slater's considerations about presence; see Slater, 2018, p. 432). Nevertheless, participants and players are usually willing to temporarily accept the new environment as the primary source of sensory information (cf. PEFR mentioned above; Wirth et al., 2007) and estimate how close to reality the environment is being experienced. Typical reactions like “the visuals of this game did not impress me much” or “it felt almost real” reflect this gradual sense of perceived realism of the virtual world. Importantly, this label of the VE is not solely determined by the virtual world itself but depends on the individual characteristics of the user and situational aspects (see section Presence as Perceived Realism).

In summary, “being there” refers to the sensation of being surrounded by a VE, with attentional resources allocated toward that environment (cf. Figure 1).

### Presence as Perceived Realism

Here, our focus is on presence in VEs. VEs create a virtual world that is supposed to be realistic and believable. Absorbing the user in VE is the easy part. It is simply achieved by blocking external stimuli and expose the user to visual sensory input provided by the HMD, which is the dominant source of sensory information. However, having a sense of being there does not necessarily imply a high level of perceived realism and, thus, a high level of presence. For example, a non-realistic world filled with simple geometrical shapes can elicit the feeling of being there, simply because it is the only environment providing sensory stimulation. Even though the attentional focus is directed to the VE in this case, one does arguably not perceive it as highly realistic, coherent, or believable. In our opinion, this problem is demonstrated by a recent study, which found that a plausible VE does not lead to higher presence ratings compared to a non-plausible VE (Hofer et al., 2020). The authors conclude that plausibility violations are not that crucial for a VR experience. It is possible, however, that the operationalization of presence (Spatial Presence Experience Scale SPES, Hartmann et al., 2015a), which measured presence as being there, is responsible for the fact that no difference occurred. Due to the HMD, the participants were fully surrounded by the virtual world in the non-plausible and in the plausible environment. Thus, it is not surprising that their subjective assessment of being there was the same. Without the assessment of perceived realism, the absence of plausibility remains unnoticed. This is why we suggest a two-dimensional operationalization of presence that includes perceived realism as an important dimension that is needed for a more complete understanding of presence.

We define *perceived realism* as the user's individual judgment about the degree of realism of the VE, in terms of (1) virtual objects, sounds and scenes, (2) credibility and plausibility of the story and its characters, and (3) naturalness and ease of the interaction with the VE. This is a new definition which we propose and which will have to be tested and, if necessary, adapted in future research. It has to be pointed out that perceived realism is not a mere function of the technical properties of the VR system but rather results from the interaction between individual expectations and technical properties such as tracking quality and visual appearance of objects. For example, the more natural the way of interacting with the VE (e.g., being able to physically walk instead of using controllers), the more realistic it appears, usually. This corresponds with those definitions that include possible actions as a relevant aspect of presence experiences (cf. Schubert et al., 1999; Sanchez-Vives and Slater, 2005; Wirth et al., 2007). But some people prefer having simple controls while others enjoy the possibility of having more advanced controls (tracking each finger separately, for example). Similarly, users typically compare the look of virtual objects to real world objects and judge the level of congruence (e.g., textures, shades, proportions, or number of details; Sutcliffe and Gault, 2004). This results in a judgment about the credibility of the VE. But a VE does not need to depict a real-life scenario in all cases. An experience could involve extremely large strawberries in a field, for example. These strawberries may not be congruent with their real counterparts in terms of size and shape, but if the user is able to touch and smell them and if the felt properties confirm that they are indeed strawberries, the user perceives the scenery as real. Accordingly, Flavián et al. (2021) showed that including the sense of a pleasant smell is not *per se* effective to increase sensory stimulation, but only if it is congruent with the virtual scenario.

Narrative elements (e.g., the story line or the historical embedding of the narrative) and characters (e.g., their fidelity or actions) are also evaluated in terms of consistency and plausibility (cf. suspension-of-disbelief; Park et al., 2010; Gorini et al., 2011). Here, too, it is not necessary that the story is close to real life. The story could involve fantasy characters or depict elements that are impossible, like traveling with lightspeed. In this regard, VR experiences share similarities with abstract art, which is often far from being close to reality. The viewer can nevertheless enjoy the piece, pick up underlying themes and expressions, interpret the artist's intentions behind the painting and spot references such as color allegories. Abstract art is well able to involve viewers in an alternative reality and thus to induce enjoyment and satisfaction. Thus, abstraction and realism are not polar opposites but can go hand in hand. A VR user can perceive an abstract environment as realistic just as a viewer of art is entangled in the world presented by the painting. Admittedly, VR still is a comparatively new technology and its full range of effects is not fully explored. This is in contrast with illustrative art, which itself has been part of human lives for thousands of years and whose effects on the viewer have been studied and interpreted by experts and art enthusiasts for centuries. It is thus conceivable that the mechanisms behind liking and enjoying art are much better understood than the impact VR can have on humans. Nevertheless, the same basic principles apply to abstract sceneries in art and in VR. Such an experience can create an alternative reality that may be far away from the reality we live in but still be consistent in itself, contain plausible analogies (e.g., the link between cause and effect) and possess a meaningful storyline (logical, involving). The term *perceived realism* emphasizes this constructive process behind the creation of one's own temporary reality and is an important part of the user's individual sense of presence. Judgments about credibility and plausibility are also likely to depend on individual differences between users (cf. *immersive tendency*). Empirical findings support this idea, as they show that presence is modulated by individual expectations, personal relevance, and personality traits (Bucolo, 2004; Weibel et al., 2010, 2011a,b,c). This, again, stresses the idea that entering VR is an active process of constructing one's own temporary reality.

In contrast to being there, perceived realism was not an essential part of influential theories of presence. Although realism is often mentioned, it is usually only regarded as a beneficial factor for the feeling of absorption. Perceived realism shares similarities with Slater's concept of Psi (2003; 2009). Psi is the extent to which one experiences the illusion of something happening as “really happening” (Slater, 2009, p. 3553). Psi occurs if there is a correlation between the user's actions and corresponding events in the VE. However, we strongly suggest that, in contrast to Psi, perceived realism is not just a perceptual illusion (cf. Slater, 2009), but rather the result of a conscious evaluation of the credibility and realness of the VE. As such, perceived realism takes into account personal experience and preferences. As an example, an experienced VR enthusiast will likely disagree with the judgment of a first-time user on a visually stunning yet otherwise unsophisticated VE (e.g., a realistic depiction of a historic site without the ability to interact with and explore the site).

Taken together, we define perceived realism as the user's individual judgment of the degree of realism of a virtual world (cf. Figure 1). This judgment is influenced by the nature of virtual objects, sounds, or scenes as well as by the plausibility of a story and its characters. We assume that the judgment about the interaction with the VE is a further factor that affects perceived realism.

### The Benefit of a Two-Dimensional Conceptualization

Perceived realism and attention are often mistakenly blended into the term being there. Lombard and Ditton (1997), for example, assume that an illusion of non-mediation is more likely to occur when perceived realism is high. This suggests a correlation between being there and perceived realism. While this is possible for traditional media like movie theaters and television, it does not really apply to VR environments since being there is easy to achieve. Indeed, the low priority attributed to perceived realism can partly be explained by the fact that the presence literature is largely based on research with early versions of HMDs with narrow field-of-views or non-VR media such as videos and desktop games. Presence in modern VR is different from presence experienced when exposed to traditional media. The user has no longer to close the gap between the physical place surrounding the computer screen and the environment that is displayed on the screen (cf. Slater and Wilbur, 1997).

To our knowledge, three studies were carried out to gather insights into the dimensionality that underlies the phenomenon of presence (Lessiter et al., 2001; Schubert et al., 2001; Witmer et al., 2005). These factor analyses yielded either realness (Schubert et al., 2001), naturalness (Lessiter et al., 2001), or sensory fidelity (Witmer et al., 2005) as a part of the final factorial structure. This underlines the need to consider perceived realism when conceptualizing presence. In many presence theories, some form of realism is mentioned but usually not addressed in detail (e.g., Lombard and Ditton, 1997; Witmer and Singer, 1998). Realism is, however, an integral part of separate conceptions such as *fidelity* (Alexander et al., 2005), *reality judgment* (Baños et al., 2000), *stimulus fidelity* (Stoffregen et al., 2003), *coherence* (Skarbez et al., 2017), and *perceived realism* (e.g., Busselle and Bilandzic, 2008; Rovira et al., 2009; Skarbez, 2016).

This latter strain of research has connected perceived realism to higher-order cognitive operations that influence how the VE is being experienced (Gilbert, 2016; Skarbez, 2016). It has been proposed that perceived realism directly relates to the user's sense of presence and that it reflects a higher level of cognitive processing (i.e., having meta-thoughts about one's VR experience; Hofer et al., 2020) that is different from the more automatic processing of being there (cf. the position of Slater, 2018, cited above). Whereas, being there is regarded as a necessary component of presence, subjective realism is a contributing factor that modulates the top-down experience of spatial presence (cf. Hofer et al., 2020). According to Skarbez (2016), perceived realism or—in his term—plausibility is the extent to which the VE meets the user's expectations, given the user's prior knowledge about the real world and the virtual world depicted in the VE (which could entail a fantasy world like Star Wars, which is well-established but not real; see also Gilbert, 2016). Currently, there is only little research about the effects of perceived realism in VR and the conducted studies generally show that higher realism goes along with stronger presence (e.g., Welch et al., 1996; Regenbrecht and Schubert, 2002; Krcmar et al., 2011; Skarbez, 2016), although the effect is often small and a recent study found no effect (Hofer et al., 2020). Perceived realism was usually conceived as either the sensory quality of the VE or as an objective mistake or inconsistence in the virtual scene (e.g., objects in the wrong order). As outlined above, we assume that the null result found by Hofer et al. (2020) is interesting because it is the result of the one-dimensional operationalization of presence (the experience of being there). Research about subjective realism in VE—i.e., virtual worlds that seem implausible only for certain users but not for others because of individual differences in expectations about the VE—are still rare (Bouchard et al., 2012, being a notable exception). The lack of experimental studies may reflect how presence research so far has mainly adhered to the traditional definitions of presence.

Along with the abovementioned authors, we think that the concept of (subjective) realism complements the sense of being there and that it needs to be incorporated into a comprehensive definition of presence in VR. In line with Busselle and Bilandzic, we have chosen the term *perceived realism*. However, while Busselle and Bilandzic describe perceived realism as the plausibility and coherence of a narrative, we suggest a broader definition of perceived realism that includes not only the story, but also the coherence and plausibility of the virtual world itself as well as the ease and realism of interactions with the physical components of the VE. Interaction is an important element because, unlike traditional media, storytelling in VR often requires the user to perform certain physical actions, such as opening a door.

To date, no concept of presence clearly distinguishes between being there as a form of attentional allocation and perceived realism. We claim that the almost exclusive focus on being there without separating attentional allocation and perceived realism is problematic for VR because the meaning of being there is highly ambiguous in VR, and therefore leads to confusions in participants. Unlike television, users are already *there* if equipped with a modern HMD: the entire visual field is taken in by the display, headphones are drowning external noise, and haptic devices emulate the sense of touch (cf. McMahan, 2003). Since VR devices effectively surround users with the virtual world, presence as the sense of being there is almost inevitably very high, even if a VE is not convincing at all (e.g., poor resolution). Indeed, there is no need of getting somewhere if one is there already.

This conceptual flaw is evident in questionnaires that are not properly adapted to modern VR and hinder a clear operationalization of presence because they include confusing questions. For example, it is not clear how participants are supposed to respond to an item like “how much did the visual/auditory aspects of the environment involve you?” from the Presence Questionnaire (PQ; Witmer et al., 2005). In VR, there is no external sensory information from the world other than the visual and auditory inputs provided by the VR. Yet other widely used questionnaires fail to differentiate between VR and other media experiences. The item “somehow I felt that the virtual world surrounded me” from the Igroup Presence Questionnaire (IPQ; Schubert et al., 2001) is another example. Being surrounded by the virtual world is literally inevitable. The Slater-Usoh-Steed Presence Questionnaire (SUS; Usoh et al., 2000; cf. Hein et al., 2018) uses the item, “during the time of the experience, which was strongest on the whole, your sense of being in the office space, or of being elsewhere?” For all items of this kind, it is difficult to give a conclusive answer because participants are fully surrounded by the VE, yet they know that they are in an office space. Skarbez (2016, p. 73ff) demonstrated that traditional questionnaires are still able to detect overall levels of presence when realism was manipulated but also suggests that the realism component itself was not captured well by the PQ and the SUS.

In fact, participants in our own VR studies regularly report difficulties when filling out presence questionnaires (e.g., Weber et al., 2019, 2020a,b). They often wonder whether they are expected to answer, “I totally agree” because a VE perceptually surrounded them or whether they are supposed to answer, “I totally disagree” because the VE was not realistic and they were at any moment in time aware of the office space surrounding them despite not seeing it. The ambiguity of questionnaire items instills different response strategies in presence questionnaires, which is highly undesirable for research. As Weibel et al. (2011c) state, “almost every empirical study on presence includes subjective data in terms of questionnaires” (p. 866). Interestingly, this also applies to the studies conducted in VR, and this is highly problematic. Presence—measured by questionnaires—is often used as a covariate in VR experiments. Yet it is not clear whether the existing questionnaires, in fact, measure what is intended: is it the allocated attention to the VE that is expected to affect the output variable or is it the judgment about the realism of the VE? Depending on the research context, it could be only one of the two dimensions or both.

The interpretation of items could be markedly improved by separating between items measuring the sense of being there as an attentional focus on the one hand, and items measuring the perceived realism of the VE on the other hand. This would allow for a clearer operationalization of presence and more valid measurements. In our own research, we have often observed that the high absorption capacity of VR has led to a ceiling effect in presence measurements. The focus on being there neglects important aspects of the virtual world and leads to ambiguities and a reduced range of possible responses in questionnaires. If perceived realism is included as a separate dimension, we assume that response ambiguities can be prevented and the variance of presence measurements will be increased. Furthermore, with the separation of being there and perceived realism, questionnaires could more easily be adapted to different forms of media and allow to better differentiate between immersive and non-immersive experiences across media.

It is noteworthy that both, the PQ and IPQ, already include a realism component (labeled “realness” and “sensory fidelity”). However, we seek for a new, substantially advanced, concept of realism that goes beyond quality assessments of the VE's sensual properties. A judgment about the plausibility of the virtual world is lacking. A graphically unsophisticated world does not necessarily go along with a low presence measure but can very well be experienced as realistic in certain scenarios. The other way around, a visually stunning experience does not necessarily lead to high presence. Presence always depends on the user's individual predispositions and situational aspects, aspects that are not reflected in current presence questionnaires (see Brackney and Priode, 2017, p. E72 for a discussion about this aspect with regard to the realism subscale of the PQ). Such aspects require new questionnaire items (e.g., “to what extent did the virtual environment meet your expectations concerning the realism of virtual objects?” “did the virtual experience provide a believable and coherent story for you?”) or completely new questionnaire formats. Additionally, the PQ specifically lacks items about the realism of the storyline and characters and instead focuses mainly on (visual) sensory fidelity. As for the IPQ, the items that measure realism are the following: “How real did the virtual world seem to you?” “How much did your experience in the virtual environment seem consistent with your real world experience?” “How real did the virtual world seem to you?” and “The virtual world seemed more realistic than the real world.” At first glance, these items look straightforward but they require the user to give a judgment about the *objective* realness of the experience instead of the user's *perceived* realness. Objective realism rather refers to system immersion in Slater's (2003; 2009) sense. Since the consensus is that presence is a subjective experience (e.g., Heeter, 1992), measuring subjective or—as we name it—perceived realism is essential for determining the degree of presence in VR.

Presence in VR positively affects various output measures such as task performance and therapy outcome (e.g., Ragan et al., 2010; Riva et al., 2015). Thus, evoking presence is essential for VR development as well as for research. It is not surprising that presence has been identified as a design ideal for synthetic environments (e.g., Draper et al., 1998). Being there is undoubtedly an essential part of the presence experience. It is especially important for traditional media such as desktop games and movies where one's senses are not completely surrounded by the medium and the user has to actively focus on the mediated world. In some cases, the feeling of being there might even be the main source of presence. In VR, however, where one quite literally enters an alternative physical reality, not much is needed to create a strong sense of being there and, thus, credible, and convincing VEs play a much more important role in achieving strong feelings of presence.

Taken together, we claim that presence in VR is more than just being absorbed by a mediated world; it depends on believable and credible as well as plausible and coherent virtual surroundings that constitute the virtual world. In our view, the feeling of presence occurs if a mediated environment (1) captures and maintains our attention and (2) is perceived as realistic. Therefore, we suggest dividing presence into the two dimensions *being there* and *perceived realism*. This is illustrated in Figure 1.

## Discussion

Current research on presence does either not consider perceived realism or a distinction between perceived realism and being there is widely missing. This resulted not only in contradictory definitions of presence but also in numerous confusing questionnaire items. Unlike other media, VR creates a highly absorbing experience and evoking a sense of being there is almost automatically achieved by rendering potentially competing external sensory input unavailable. Indeed, creating credible and realistic VEs is crucial for achieving strong feelings of presence in VR. Enhancing the perceived realism of VEs is a main focus of VR development. Yet, it is often neglected in theoretical conceptions or blended in with being there as a form of attentional allocation. Perceived realism is especially relevant for VR experiences, not only because they effectively block external sensory input but also for the better use of the multi-sensory stimulation that is possible with advanced technology. VR experiences have already been enhanced with the sense of touch (e.g., Rietzler et al., 2018), weight (e.g., Zenner and Krüger, 2017), and even smell (e.g., Flavián et al., 2021; this paper also provides an up-to-date overview of multisensory research). There are devices which let users naturally walk through the VE on a treadmill (cf. Wehden et al., 2021) or with specially designed shoes (e.g., Reinhardt et al., 2019). Additionally, haptic gloves or even whole body tracking suits have emerged (e.g., Loughlan, 2017). Already since the early days of immersive media, the goal was to stimulate as many senses as possible to create a sense of reality (cf. Heilig, 1992, as an early example). This goes far beyond traditional media which are not designed to directly emulate sensory input. Therefore, the definition of presence in VR is significantly different from other media and requires a new approach that emphasizes the importance of perceived realism.

Speaking of all the technological advancements in VR, however, there is also a significant caveat to keep in mind: simply combining multiple sensory simulators is no guarantee to achieve a high sense of presence. With more elaborate equipment there are also higher expectations toward the technology that raise the bar for effectively perceiving realism. Including the smell and taste of an object is of no use if the experience of touching it does not keep up with the other stimulations (an example being the study from Bennett and Stevens, 2005; see also Culbertson and Kuchenbecker, 2016; Zhao and Follmer, 2018). An apple that smells like an apple but feels like a piece of cardboard may be enough to irritate a user and disrupt perceived reality.

An interesting example where a new, VR-specific definition of a research concept has successfully been established, is provided by the three sub-components model of the *sense of embodiment*, introduced by Kilteni et al. (2012). According to the authors, experiencing the same sensations toward a virtual body as to one's own biological body requires a sense of *self-location* (the virtual body's position is congruent with one's own position), a sense of *agency* (the virtual body's actions correspond to my own actions), and a sense of *body ownership* (the virtual body feels and looks like my own body). This concept successfully expands the definition of embodiment, which has been used for explaining the effect of accepting an artificial limb as a part of one's own body (cf. *rubber-hand illusion*; Botvinick and Cohen, 1998). Having a sense of agency together with a realistic depiction of a limb which is located at the same position as one's own limb is effectively only possible in VR. Advanced studies in body ownership used a combination of clues in VR to successfully embody participants in artificial bodies or body parts (e.g., Jo et al., 2017). This rapid development required a new and advanced definition of the concept of embodiment, which resulted in the three sub-components model, mentioned above. This approach has since gained popularity in the research community and is now regularly used to define embodiment in recent studies with virtual bodies (cf. Fribourg et al., 2020). The situation with embodiment is thus comparable to that of presence. Here, too, we believe that technological development has led to a situation where the research community could benefit from a refined definition. We even go one step further and claim that the study of presence in VR only makes sense if the concept is in fact redefined. Here we propose perceived realism as a new and separate dimension of presence.

Including questions about the perceived realism of a VE and separating them from questions asking about attentional allocation could reduce the confusion in presence questionnaires. To our knowledge, the distinction of two dimensions of presence will provide a clearer operationalization, which will make effects of presence on other measures better interpretable. Like the sense of embodiment, which has been proposed to be split into the components sense of self-location, sense of agency, and sense of body ownership, presence needs to be assessed with sub-components that together form the overall sense of presence: being there and perceived realism. This will ultimately close the gap between developers' main concerns about presence (perceived realism) and the researchers' focus on presence (being there). As mentioned, it is not only important to measure the realism associated with each sense being stimulated. It also necessary to keep track of possible interactions between them and ask the user about his integrated sense of perceived realism.

A compelling virtual world needs to be believable, authentic, and visually appealing in addition to capturing attention. This is especially true for professional VR applications that rely on maximal comparability to the real world, such as training environments for surgeons, firefighters, or pilots. A clearer separation of being there and perceived realism makes presence more generalizable across media and especially more applicable to VR. In terms of VR, questionnaires need to focus on the perceived realism of the VE. This way, game designers could obtain subjective quality measures of VEs. Researchers could use more detailed presence measures that separate being there and perceived realism. This will lead to stronger and clearer results and strengthen the correlations between presence and different output variables such as enjoyment, therapy outcome, or learning.

This opinion paper aims to initiate a discussion about what constitutes presence in VR. We have proposed a plausible definition that is based on existing findings. Nevertheless, in a first step, future research is needed to challenge this definition.

An evaluation of the proposed definition could be accomplished by developing a new questionnaire that incorporates relevant aspects in the context of perceived realism, e.g., the perception of virtual objects, sounds and scenes; plausibility of the story; naturalness and ease of interactions. As part of the questionnaire construction, an appropriate item pool would need to be generated to map these aspects (for an overview on scale development see for example Irwing et al., 2018). Combined with existing questions that depict presence as being there, the dimensionality of presence in VE could be extracted and it could be tested whether the dimensions we propose can be confirmed empirically. If so, it would be interesting to investigate whether being there and perceived realism are orthogonal dimensions of an overall presence construct or whether they are correlated.

A new questionnaire would help to learn more about (1) the extent to which being there and perceived realism influence possible outcome variables and to assess (2) which dimension explains more variance. For this purpose, it would be promising to conduct an experiment in which being there (high vs. low) and perceived realism (high vs. low) are manipulated in a 2×2 design. This could help to find out more about the relative influence of the two dimensions. Below we outline such an experiment. It is a suggestion that might be useful to find out the influence that the two dimensions can exert. The idea is to transfer a well-known conventional video game, which involves simple graphics yet is fun to play, into VR. A good example could be *Tetris* (Pajitnov, 1985), but also any other game involving simple geometrical shapes could work; another idea is the use of puzzle games, where there already exist several examples of sophisticated and realistic VR implementations (e.g., The Talos Principle, 2015; for an overview on engaging games see Prensky, 2001). These games are very popular since they involve challenging tasks, they provide immediate performance feedback and ways to directly interact with the virtual world and thus, they can be adapted to either a highly involving or a completely uninvolving VR experience. This in turn allows to manipulate the dimension “being there”: The condition *high level of being there* could consist of an exciting version of the game (involving challenges and feedback on one's performance) so that attentional resources are bound by the VE. The condition *low level of being there* is a version of the same game, but in a version that is not involving at all with the result that the attentional resources are not allocated toward the VE; this could for example be an overly simple and unentertaining version of the game, perhaps in combination with a repetitive and unchallenging task. The factor perceived realism could be varied as follows: in the condition *high level of perceived realism*, the game could be embedded in an overall story (where the player is on track to solve a mystery or rescue another character, etc.) and with more emphasis on a realistic experience in terms of high vividness, refined visual scenes and the involvement of multiple sensory cues (haptic feedback when touching the shapes, shapes appear in a realistic amusement-park-like scenario, etc.). In contrast, the condition *low level of perceived realism* should not involve any (logically structured) storyline, characters, or surrounding environment and the environment could look Minecraft-like (cf. Minecraft, 2011) with low-resolution. Of course, rigorous study demands that only a few characteristics are manipulated at once and surely, a whole series of experiments will be needed to test all of these contributing factors.

We are convinced that a more thorough assessment of the VR experience will be beneficial for research and applications alike. VE's are now rapidly gaining importance, and it is by all means necessary to better understand the dimensions that are involved in order to exploit VR's full potential, and also further develop the technology so that it serves society best.

## Author Contributions

SW, DW, and FM contributed to the conception of the manuscript. SW wrote the first draft of the manuscript. All authors contributed to manuscript revision, read, and approved the submitted version.

## Conflict of Interest

The authors declare that the research was conducted in the absence of any commercial or financial relationships that could be construed as a potential conflict of interest.

## References

[B1] Alexander A. L. Brunyé T. Sidman J. Weil S. A. (2005). From Gaming to Training: A Review of Studies on Fidelity, Immersion, Presence, and Buy-in and Their Effects on Transfer in PC-Based Simulations and Games. Woburn, MA: DARWARS Training Impact Group, 1–14.

[B2] Baños R. M. Botella C. García-Palacios A. Villa H. Perpiñá C. Alcaniz M. (2000). Presence and reality judgment in virtual environments: a unitary construct? Cyberpsychol. Behav. 3, 327–335. 10.1089/10949310050078760

[B3] Baños R. M. Botella C. García-Palacios A. Villa H. Perpiñá C. Gallardo M. (1999). Psychological variables and reality judgment in virtual environments: the roles of absorption and dissociation. Cyberpsychol. Behav. 2, 143–148. 10.1089/cpb.1999.2.143 19178250

[B4] Bennett E. Stevens B. (2005). The effect that touching a projection augmented model has on object-presence, in Ninth International Conference on Information Visualisation (IV'05) (London), 790–795.

[B5] Botvinick M. Cohen J. (1998). Rubber hands “feel” touch that eyes see. Nature 391, 756–756. 10.1038/35784 9486643

[B6] Bouchard S. Dumoulin S. Talbot J. Ledoux A. A. Phillips J. Monthuy-Blanc J.. (2012). Manipulating subjective realism and its impact on presence: preliminary results on feasibility and neuroanatomical correlates. Interact. Comput. 24, 227–236. 10.1016/j.intcom.2012.04.011

[B7] Bracken C. C. Skalski P. (2010). Immersed in Media: Telepresence in Everyday Life. New York, NY: Rougledge.

[B8] Brackney D. E. Priode K. (2017). Back to reality: the use of the presence questionnaire for measurement of fidelity in simulation. J. Nurs. Meas. 25, 66E−73E. 10.1891/1061-3749.25.2.E66 28789741

[B9] Brown E. Cairns P. (2004). A grounded investigation of game immersion, in CHI'04 Extended Abstracts on Human Factors in Computing Systems (Vienna: ACM), 1297–1300.

[B10] Bucolo S. (2004). Understanding cross cultural differences during interaction within immersive virtual environments, in Proceedings of the 2004 ACM SIGGRAPH International Conference on Virtual Reality Continuum and Its Applications in Industry (New York, NY: ACM), 221–224.

[B11] Busselle R. Bilandzic H. (2008). Fictionality and perceived realism in experiencing stories: a model of narrative comprehension and engagement. Commun. Theory 18, 255–280. 10.1111/j.1468-2885.2008.00322.x

[B12] Bystrom K. E. Barfield W. Hendrix C. (1999). A conceptual model of the sense of presence in virtual environments. Presence Teleoper. Virtual Environ. 8, 241–244. 10.1162/105474699566107

[B13] Culbertson H. Kuchenbecker K. J. (2016). Importance of matching physical friction, hardness, and texture in creating realistic haptic virtual surfaces. IEEE Trans. Haptics 10, 63–74. 10.1109/TOH.2016.2598751 28328499

[B14] Curran N. (2018). Factors of Immersion. Wiley Handb. Hum. Comput. Interact. 1, 239–254. 10.1002/9781118976005.ch13

[B15] Draper J. V. Kaber D. B. Usher J. M. (1998). Telepresence. Hum. Factors 40, 354–375. 10.1518/001872098779591386 9849099

[B16] Flavián C. Ibáñez-Sánchez S. Orús C. (2021). The influence of scent on virtual reality experiences: the role of aroma-content congruence. J. Bus. Res. 123, 289–301. 10.1016/j.jbusres.2020.09.036

[B17] Fribourg R. Argelaguet F. Lécuyer A. Hoyet L. (2020). Avatar and sense of embodiment: studying the relative preference between appearance, control, and point of view. IEEE Trans. Vis. Comput. Graph. 26, 2062–2072. 10.1109/TVCG.2020.2973077 32070975

[B18] Gilbert S. B. (2016). Perceived realism of virtual environments depends on authenticity. Presence Teleoper. Virtual Environ. 24, 322–324. 10.1162/PRES_a_00276

[B19] Gorini A. Capideville C. S. De Leo G. Mantovani F. Riva G. (2011). The role of immersion and narrative in mediated presence: the virtual hospital experience. Cyberpsychol. Behav. Soc. Netw. 14, 99–105. 10.1089/cyber.2010.0100 20649451

[B20] Grassini S. Laumann K. (2020). Questionnaire measures and physiological correlates of presence: a systematic review. Front. Psychol. 11:349. 10.3389/fpsyg.2020.00349 32265769 PMC7096541

[B21] Hartmann T. Wirth W. Schramm H. Klimmt C. Vorderer P. Gysbers A.. (2015a). The spatial presence experience scale (SPES). J. Media Psychol. 28, 1–15. 10.1027/1864-1105/a000137

[B22] Hartmann T. Wirth W. Vorderer P. Klimmt C. Schramm H. Böcking S. (2015b). Spatial presence theory: state of the art and challenges ahead, in Immersed in Media, eds Lombard M. Biocca F. Freeman J. IJsselsteijn W. Schaevitz R. (Cham: Springer), 115–135.

[B23] Heeter C. (1992). Being there: the subjective experience of presence. Presence Teleoper. Virtual Environ. 1, 262–271. 10.1162/pres.1992.1.2.262

[B24] Heilig M. L. (1992). El cine del futuro: the cinema of the future. Presence Teleoper. Virtual Environ. 1, 279–294. 10.1162/pres.1992.1.3.279

[B25] Hein D. Mai C. Hußmann H. (2018). The usage of presence measurements in research: a review, in Proceedings of the International Society for Presence Research Annual Conference (Presence) (Kyoto: The International Society for Presence Research).

[B26] Hofer M. Hartmann T. Eden A. Ratan R. Hahn L. (2020). The role of plausibility in the experience of spatial presence in virtual environments. Front. Virtual Real. 1:2. 10.3389/frvir.2020.00002

[B27] Irwing P. Booth T. Hughes D. J. (2018). The Wiley Handbook of Psychometric Testing: A Multidisciplinary Reference on Survey, Scale. and Test Development. Hoboken, NJ: Wiley.

[B28] Jaalama K. Fagerholm N. Julin A. Virtanen J. P. Maksimainen M. Hyypp,ä H. (2021). Sense of presence and sense of place in perceiving a 3D geovisualization for communication in urban planning–differences introduced by prior familiarity with the place. Landsc. Urban Plan. 207:103996. 10.1016/j.landurbplan.2020.103996

[B29] Jo D. Kim K. Welch G. F. Jeon W. Kim Y. Kim K. H.. (2017). The impact of avatar-owner visual similarity on body ownership in immersive virtual reality, in Proceedings of the 23rd ACM Symposium on Virtual Reality Software and Technology (New York; NY), 1–2.

[B30] Kilteni K. Groten R. Slater M. (2012). The sense of embodiment in virtual reality. Presence Teleoper. Virtual Environ. 21, 373–387. 10.1162/PRES_a_00124

[B31] Kim T. Biocca F. (1997). Telepresence via television: two dimensions of telepresence may have different connections to memory and persuasion. J. Comp. Mediat. Commun. 3:JCMC325. 10.1111/j.1083-6101.1997.tb00073.x

[B32] Krcmar M. Farrar K. McGloin R. (2011). The effects of video game realism on attention, retention, and aggressive outcomes. Comput. Hum. Behav. 27, 432–439. 10.1016/j.chb.2010.09.005

[B33] Lee K. M. (2004). Presence, explicated. Commun. Theory 14, 27–50. 10.1111/j.1468-2885.2004.tb00302.x

[B34] Lessiter J. Freeman J. Keogh E. Davidoff J. (2001). A cross-media presence questionnaire: the ITC-sense of presence inventory. Presence Teleoper. Virtual Environ. 10, 282–297. 10.1162/105474601300343612

[B35] Lombard M. Ditton T. (1997). At the heart of it all: the concept of presence. J. Comp. Med. Commun. 3:JCMC321. 10.1111/j.1083-6101.1997.tb00072.x

[B36] Loughlan J. (2017). News briefing-mobile world congress: virtual reality-5G headset coupled with full-body suit promises complete virtual immersion. Eng. Technol. 12, 13–13. 10.1049/et.2017.0314

[B37] McMahan A. (2003). Immersion, engagement, and presence, in The Video Game Theory Reader, eds Wolf M. J. P. Perron B. (New York, NY: Routledge, Taylor and Francis Group), 77–78.

[B38] Minecraft (2011). [Video game]. Stockholm, Sweden: Mojang Studios. Available online at: https://minecraft.net/en-us/ (Retrieved April 5, 2021).

[B39] Minsky M. (1980). Telepresence. Omni 2, 45–51.

[B40] Nilsson N. C. Nordahl R. Serafin S. (2016). Immersion revisited: a review of existing definitions of immersion and their relation to different theories of presence. Hum. Technol. 12, 108–134. 10.17011/ht/urn.201611174652

[B41] Pajitnov A. (1985). Tetris [Video game]. Hawaii: Tetris Holding LLC. Available online at: https://tetris.com/ (Retrieved April 5, 2021).

[B42] Park N. Lee K. M. Jin S. A. A. Kang S. (2010). Effects of pre-game stories on feelings of presence and evaluation of computer games. Int. J. Hum. Comput. Stud. 68, 822–833. 10.1016/j.ijhcs.2010.07.002

[B43] Prensky M. (2001). Fun, play and games: What makes games engaging, in Digital Game-based Learning, ed Prensky M. (New York, NY: McGraw-Hill).

[B44] Ragan E. D. Sowndararajan A. Kopper R. Bowman D. A. (2010). The effects of higher levels of immersion on procedure memorization performance and implications for educational virtual environments. Presence Teleoper. Virtual Environ. 19, 527–543. 10.1162/pres_a_00016

[B45] Rauscher M. (2021). Virtual reality in tourism: is it “Real” enough? Acad. Turistica Tour. Innov. J. 13, 127–138. 10.26493/2335-4194.13.127-138

[B46] Regenbrecht H. Schubert T. (2002). Real and illusory interactions enhance presence in virtual environments. Presence Teleoper. Virtual Environ. 11, 425–434. 10.1162/105474602760204318

[B47] Reinhardt J. Lewandowski E. Wolf K. (2019). Build your own! open-source VR shoes for unity3d, in Proceedings of the 10th Augmented Human International Conference 2019 (New York, NY), 1–2.

[B48] Rietzler M. Geiselhart F. Frommel J. Rukzio E. (2018). Conveying the perception of kinesthetic feedback in virtual reality using state-of-the-art hardware, in Proceedings of the 2018 CHI Conference on Human Factors in Computing Systems (Montreal, QC), 1–13.

[B49] Riva G. Botella C. Baños R. Mantovani F. García-Palacios A. Quero S.. (2015). Presence-inducing media for mental health applications, in Immersed in Media, eds Lombard M. Biocca F. Freeman J. IJsselsteijn W. Schaevitz R. (Cham: Springer), 283–332.

[B50] Rovira A. Swapp D. Spanlang B. Slater M. (2009). The use of virtual reality in the study of people's responses to violent incidents. Front. Behav. Neurosci. 3:59. 10.3389/neuro.08.059.2009 20076762 PMC2802544

[B51] Sanchez-Vives M. V. Slater M. (2005). From presence to consciousness through virtual reality. Nat. Rev. Neurosci. 6, 332–339. 10.1038/nrn1651 15803164

[B52] Schubert T. Friedmann F. Regenbrecht H. (1999). Embodied presence in virtual environments, in Visual Representations and Interpretations, eds Paton R. Neilson I. (London: Springer), 269–278.

[B53] Schubert T. Friedmann F. Regenbrecht H. (2001). The experience of presence: factor analytic insights. Presence Teleoper. Virtual Environ. 10, 266–281. 10.1162/105474601300343603

[B54] Schuemie M. J. Van Der Straaten P. Krijn M. Van Der Mast C. A. (2001). Research on presence in virtual reality: a survey. Cyberpsychol. Behav. 4, 183–201. 10.1089/109493101300117884 11710246

[B55] Sheridan T. B. (1992). Musings on telepresence and virtual presence. Presence Teleoper. Virtual Environ. 1, 120–126. 10.1162/pres.1992.1.1.120

[B56] Skarbez R. (2016). Plausibility illusion in virtual environments (Doctoral dissertation). The University of North Carolina at Chapel Hill, Chapel Hill, NC, United States.

[B57] Skarbez R. Brooks F. P. Jr Whitton M. C. (2017). A survey of presence and related concepts. ACM Comput. Surv. 50:96. 10.1145/3134301

[B58] Slater M. (2003). A note on presence terminology. Presence Connect 3, 1–5. Available online at: http://citeseerx.ist.psu.edu/viewdoc/download?doi=10.1.1.800.3452&rep=rep1&type=pdf

[B59] Slater M. (2009). Place illusion and plausibility can lead to realistic behaviour in immersive virtual environments. Philos. Trans. R. Soc. B Biol. Sci. 364, 3549–3557. 10.1098/rstb.2009.0138 19884149 PMC2781884

[B60] Slater M. (2018). Immersion and the illusion of presence in virtual reality. Br. J. Psychol. 109, 431–433. 10.1111/bjop.12305 29781508

[B61] Slater M. Steed A. (2000). A virtual presence counter. Presence Teleoper. Virtual Environ. 9, 413–434. 10.1162/105474600566925

[B62] Slater M. Wilbur S. (1997). A framework for immersive virtual environments (FIVE): speculations on the role of presence in virtual environments. Presence Teleoper. Virtual Environ. 6, 603–616. 10.1162/pres.1997.6.6.603

[B63] Steuer J. (1992). Defining virtual reality: dimensions determining telepresence. J. Commun. 42, 73–93. 10.1111/j.1460-2466.1992.tb00812.x

[B64] Stoffregen T. A. Bardy B. G. Smart L. J. Pagulayan R. J. (2003). On the nature and evaluation of fidelity in virtual environments, in Virtual and Adaptive Environments: Applications, Implications, and Human Performance Issues, eds Hettinger L. J. Haas M. W. (Mahwah, NJ: Lawrence Erlbaum Associates Publishers), 111–128.

[B65] Sutcliffe A. Gault B. (2004). Heuristic evaluation of virtual reality applications. Interact. Comput. 16, 831–849. 10.1016/j.intcom.2004.05.001

[B66] The Talos Principle (2015). [Video game]. Austin, TX: Devolver Digital. Available online at: http://www.croteam.com/talosprinciple/ (Retrieved April 5, 2021).

[B67] Usoh M. Catena E. Arman S. Slater M. (2000). Using presence questionnaires in reality. Presence Teleoper. Virtual Environ. 9, 497–503. 10.1162/105474600566989

[B68] Weber S. Mast F. W. Weibel D. (2019). Body size illusions influence perceived size of objects: a validation of previous research in virtual reality. Virtual Real. 24, 1–13. 10.1007/s10055-019-00402-z

[B69] Weber S. Mast F. W. Weibel D. (2020a). Experiencing presence in a gaming activity improves mood after a negative mood induction. Int. J. Gaming Comput. Mediat. Simul. 12, 1–22. 10.4018/IJGCMS.2020100101

[B70] Weber S. Weibel D. Mast F. (2020b). How self-motion in virtual reality affects the subjective perception of time. Timing Time Percept. 8, 119–136. 10.1163/22134468-20191152

[B71] Weech S. Kenny S. Barnett-Cowan M. (2019). Presence and cybersickness in virtual reality are negatively related: a review. Front. Psychol. 10:158. 10.3389/fpsyg.2019.00158 30778320 PMC6369189

[B72] Wehden L. O. Reer F. Janzik R. Tang W. Y. Quandt T. (2021). The slippery path to total presence: how omnidirectional virtual reality treadmills influence the gaming experience. Media Commun. 9, 5–16. 10.17645/mac.v9i1.3170

[B73] Weibel D. Wissmath B. (2011). Immersion in computer games—the role of spatial presence and flow. Int. J. Comput. Games Technol. 1–14:282345. 10.1155/2011/282345

[B74] Weibel D. Wissmath B. Mast F. W. (2010). Immersion in mediated environments: the role of personality traits. Cyberpsychol. Behav. Soc. Netw. 13, 251–256. 10.1089/cyber.2009.0171 20557243

[B75] Weibel D. Wissmath B. Mast F. W. (2011a). Influence of mental imagery on spatial presence and enjoyment assessed in different types of media. Cyberpsychol. Behav. Soc. Netw. 14, 607–612. 10.1089/cyber.2010.0287 21352082

[B76] Weibel D. Wissmath B. Mast F. W. (2011b). The role of cognitive appraisal in media-induced presence and emotions. Cogn. Emot. 25, 1291–1298. 10.1080/02699931.2010.543016 21432638

[B77] Weibel D. Wissmath B. Stricker D. (2011c). The influence of neuroticism on spatial presence and enjoyment in films. Pers. Individ. Dif. 51, 866–869. 10.1016/j.paid.2011.07.01121352082

[B78] Welch R. B. Blackmon T. T. Liu A. Mellers B. A. Stark L. W. (1996). The effects of pictorial realism, delay of visual feedback, and observer interactivity on the subjective sense of presence. Presence Teleoper. Virtual Environ. 5, 263–273. 10.1162/pres.1996.5.3.263

[B79] Wirth W. Hartmann T. Böcking S. Vorderer P. Klimmt C. Schramm H.. (2007). A process model of the formation of spatial presence experiences. Media Psychol. 9, 493–525. 10.1080/15213260701283079

[B80] Wissmath B. Weibel D. Schmutz J. Mast F. W. (2011). Being present in more than one place at a time? Patterns of Mental Self-Localization. Conscious. Cogn. 20, 1808–1815. 10.1016/j.concog.2011.05.008 21641823

[B81] Witmer B. G. Jerome C. J. Singer M. J. (2005). The factor structure of the presence questionnaire. Presence Teleoper. Virtual Environ. 14, 298–312. 10.1162/105474605323384654

[B82] Witmer B. G. Singer M. J. (1998). Measuring presence in virtual environments: a presence questionnaire. Presence 7, 225–240. 10.1162/105474698565686

[B83] Yang T. Lai I. K. W. Fan Z. B. Mo Q. M. (2021). The impact of a 360° virtual tour on the reduction of psychological stress caused by COVID-19. Technol. Soc. 64:101514. 10.1016/j.techsoc.2020.101514 33424061 PMC7775825

[B84] Zenner A. Krüger A. (2017). Shifty: a weight-shifting dynamic passive haptic proxy to enhance object perception in virtual reality. IEEE Trans. Vis. Comp. Graphics 23, 1285–1294. 10.1109/TVCG.2017.2656978 28129164

[B85] Zhao Y. Follmer S. (2018). A functional optimization based approach for continuous 3d retargeted touch of arbitrary, complex boundaries in haptic virtual reality, in Proceedings of the 2018 CHI Conference on Human Factors in Computing Systems (Montreal, QC), 1–12.

